# Exciplex electroluminescence and photoluminescence spectra of the new organic materials based on zinc complexes of sulphanylamino-substituted ligands

**DOI:** 10.1186/1556-276X-7-206

**Published:** 2012-04-03

**Authors:** Mikhail G Kaplunov, Svetlana S Krasnikova, Sergey L Nikitenko, Natalia L Sermakasheva, Igor K Yakushchenko

**Affiliations:** 1 Institute of Problems of Chemical Physics RAS, Chernogolovka, Moscow Region 142432, Russia

## Abstract

We have investigated the electroluminescence spectra of the electroluminescent devices based on the new zinc complexes of amino-substituted benzothiazoles and quinolines containing the C-N-M-N chains in their chelate cycles. The spectra exhibit strong exciplex bands in the green to yellow region 540 to 590 nm due to interaction of the excited states of zinc complexes and triaryl molecules of the hole-transporting layer. For some devices, the intrinsic luminescence band of 460 nm in the blue region is also observed along with the exciplex band giving rise to an almost white color of the device emission. The exciplex band can be eliminated if the material of the hole-transporting layer is not a triarylamine derivative. We have also found the exciplex emission in the photoluminescence spectra of the films containing blends of zinc complex and triphenylamine material.

## Background

In typical organic light emitting devices (OLEDs), light originates from radiative relaxation of molecular excited states formed by electrons and holes injected from electrodes and localized on individual molecular sites. That is, the results are interpreted as due to Frenkel exciton generation and recombination [1,2]. In particular, this is applied to the bilayer OLEDs composed of metal 8-hydroxyquinolates Mq_3 _(M = Al, Ga, In, or Sc) as an electron-transporting and emitting layer and amines like triphenylamine derivative (TPD) as a hole-transporting layer. The electroluminescence (EL) spectra of these devices are close to the photoluminescence (PL) spectra of corresponding Mq_3 _molecules [1-3]. The similarity of the EL and PL spectra was also observed for zinc complexes with hydroxy-substituted quinolines, benzothiazoles, oxadiazoles, and related ligands [4-6].

In some bilayer devices, interactions of donor and acceptor molecules at the organic/organic interface can lead to formation of an exciplex state. Exciplex is a kind of excited state complex formed between donor and acceptor, with one in the excited state and the other in the ground state. Exciplex usually leads to the red shifted emission and broadened spectrum relative to the emissions of the individual acceptor or donor [7-10]. Exciplex formation at the solid interface between Alq_3 _and the electron-rich multiple triarylamine hole-transporting materials m-MTDATA and t-Bu-TBATA was observed in a study by Itano et al. [11].

For pure homochromatic OLEDs, exciplexes should be avoided [10,12,13]. On the other hand, exciplexes were proposed to design white OLEDs and to tune the OLED emission color [7,14-17]. One of the problems in utilizing the exciplex effects in devices is finding systems with high exciplex EL efficiency, so design of new materials and investigation of the active factors for efficient exciplex emission are a subject of significance.

**Figure 1:**
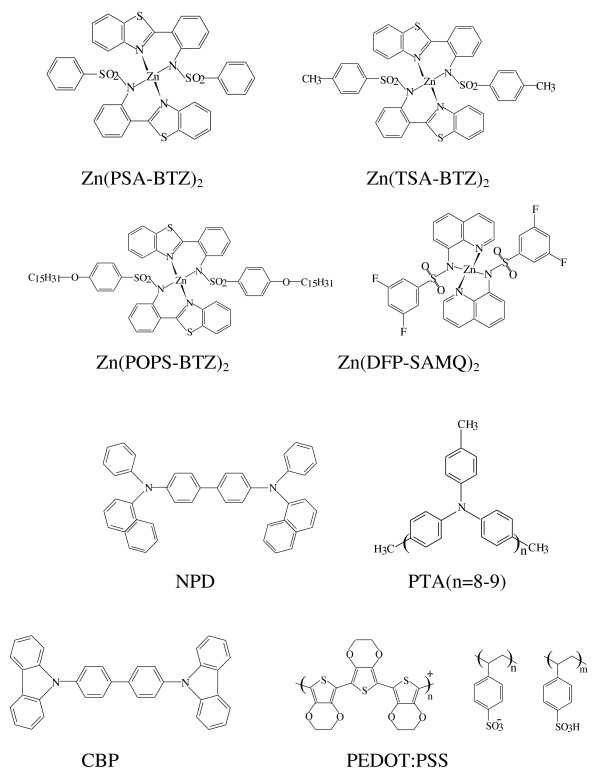
**Structures of zinc complexes and of materials for hole-transporting layers**.

In the present work, we have investigated spectral properties of the electroluminescent devices based on the novel zinc-chelate complexes of sulphanilamino-substituted quinolines and benzothiazoles [18-21]. The structures of zinc complexes are shown in Figure 1. Most presently known metal complexes used for OLEDs contain the chelate cycles including the C-O-M-N chains [1-6,9-11]. In the complexes studied here, the oxygen atom in the chelate cycles is replaced by a nitrogen atom of the sulphanylamino groups forming the C-N-M-N chains. The presence of a spatially extended, electron-rich amine segment in the zinc complex molecule can enhance its ability of intermolecular interactions with the molecules of the hole-transporting layer and hence magnify the possibility of exciplex forming.

## Methods

Electroluminescence was observed for the layered structures containing the transparent anode of the indium-tin oxide (ITO), organic hole-transporting layer, luminescence layer of one of the studied complexes, and the metallic cathode of Al:Ca (5%) alloy. The hole-transporting materials used were triaryl derivatives - well-known naphthyl-substituted benzidine derivative (NPD) and the oligomer of triphenylamine oligo(4,4'-(4''-methyl)triphenylamine) (PTA) [22]. The carbazol derivative 4,4'-bis(N-carbazolyl)-1,1'-biphenyl (CBP) and poly(3,4-ethylenedioxythiophene) poly(styrenesulfonate) (PEDOT:PSS) were also used for forming the hole-transporting layer. The structures of these compounds are shown in Figure 1. All the materials are characterized by blue PL 450 to 470 nm [18-21]. Zinc complexes and PTA were synthesized as described elsewhere [18-22]. NPD, CBP, and PEDOT:PSS were supplied by Aldrich (Sigma-Aldrich Rus LLC, Moscow, Russia). All the organic layers in the OLED devices (except PTA and PEDOT:PSS) were prepared by vacuum evaporation. PTA and PEDOT:PSS were spin casted from toluene and aqueous solutions, respectively. The EL and PL spectra were measured with the Ocean Optics fiber optics spectrometers QE65000 or PC1000 (Eurolase Ltd., Moscow, Russia). LED with *λ *= 370 nm was used to excite the PL.

We have prepared and measured the EL spectra of the following OLED devices based on zinc complexes with sulphanilamino-substituted ligands.

device 1: ITO/PTA/NPD/Zn(PSA-BTZ)_2_/Al:Ca

device 2: ITO/PTA/Zn(PSA-BTZ)_2_/Al:Ca

device 3: ITO/PTA/NPD/CBP/Zn(PSA-BTZ)_2_/Al:Ca

device 4: ITO/PTA/CBP/Zn(PSA-BTZ)_2_/Al:Ca

device 5: ITO/PEDOT:PSS/Zn(PSA-BTZ)_2_/Al:Ca

device 6: ITO/PTA/NPD/Zn(TSA-BTZ)_2_/Al:Ca

device 7: ITO/PTA/Zn(TSA-BTZ)_2_/Al:Ca

device 8: ITO/PTA/NPD/Zn(POPS-BTZ)_2_/Al:Ca

device 9: ITO/PTA/NPD/CBP/Zn(POPS-BTZ)_2_/Al:Ca

device 10: ITO/PTA/NPD/Zn(DFP-SAMQ)_2_/Al:Ca

device 11: ITO/PTA/Zn(DFP-SAMQ)_2_/Al:Ca

In some devices, both PTA and NPD deposited in succession were used as materials for hole-transporting layers. In any case, the EL spectrum of the device is determined by the hole-transporting material, which is in contact with the zinc complex. The devices are typically characterized by bias voltages of light appearance about 2.5 to 3 V and brightness of 10^3 ^cd/m^2 ^at 10 V.

## Results and discussion

### EL spectra of OLEDs based on Zn(PSA-BTZ)_2_

**Figure 2:**
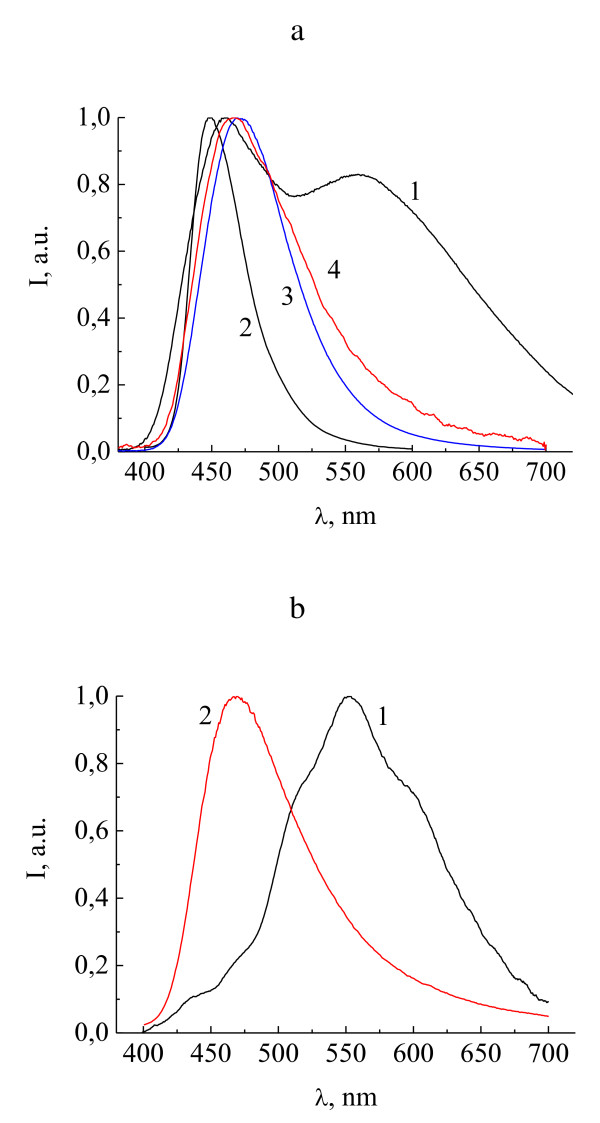
**EL spectra of Zn(PSA-BTZ)_2 _in devices 1 and 2**. **(a) **EL spectrum of device 1, ITO/PTA/NPD/Zn(PSA-BTZ)_2_/Al:Ca (1); PL spectrum of Zn(PSA-BTZ)_2 _powder (2); EL spectrum of device 3, ITO/PTA/NPD/CBP/Zn(PSA-BTZ)_2_/Al:Ca (3); and EL spectrum of device 5, ITO/PEDOT:PSS/Zn(PSA-BTZ)_2_/Al:Ca (4); **(b) **EL spectra of device 2, ITO/PTA/Zn(PSA-BTZ)_2_/Al:Ca, (1) and device 4, ITO/PTA/CBP/Zn(PSA-BTZ)_2_/Al:Ca (2).

Figure 2 shows the EL spectra of Zn(PSA-BTZ)_2 _in two electroluminescence devices: device 1, ITO/PTA/NPD/Zn(PSA-BTZ)_2_/Al:Ca, (Figure 2a, curve 1) and device 2, ITO/PTA/Zn(PSA-BTZ)_2_/AlCa, (Figure 2b, curve 1). For comparison, curve 2 in Figure 2a shows the PL spectrum of Zn(PSA-BTZ)_2 _powder. The EL spectrum of device 1 contains two bands with maxima at 460 and 560 nm. Maximum of the first band is close to that of the PL peak of Zn(PSA-BTZ)_2 _powder at 450 nm and may be attributed to the intrinsic luminescence of Zn(PSA-BTZ)_2_. The second peak may be probably due to exciplex formation between NPD and Zn(PSA-BTZ)_2_. For device 2, the EL spectrum exhibits only wide band with a maximum at 553 nm, which may be attributed to exciplex formation between PTA and Zn(PSA-BTZ)_2_.

Exciplex can be formed between the ground state of a donor molecule and the excited state of an acceptor molecule [7]. In our case, the donor molecule is presented by NPD or PTA, and the acceptor molecule by Zn(PSA-BTZ)_2 _complex. Exciplex band corresponds to the transition from the excited state of the acceptor and the ground state of the donor and has lower transition energy compared to the intrinsic emission band corresponding to the transition between the excited and ground state of the acceptor molecule [7].

The combination of narrow intrinsic band and wide exciplex band gives a very wide emission spread over the whole visible spectrum, which is a way to obtain white light emitting diodes [7,14-17,19-21]. For device 1, the CIE chromaticity coordinates are *x *= 0.31 and *y *= 0.34, which is close to that of the white light (*x *= 0.33, *y *= 0.33).

### Elimination of exciplex emission for the devices based on Zn(PSA-BTZ)_2_

To prove the exciplex origin of the long-wavelength EL, we have fabricated several control devices in which the long-wave EL bands are eliminated. One of the methods for preventing exciplex emission is the insertion of an additional layer between the hole-transporting and electron-transporting materials [9,10,12,13]. CBP is considered as one of the materials appropriate for such layers [10]. We have fabricated two control devices with CBP as the intermediate layer: ITO/PTA/NPD/CBP/Zn(PSA-BTZ)_2_/Al:Ca (device 3) and ITO/PTA/CBP/Zn(PSA-BTZ)_2_/Al:Ca (device 4). Figure 2 shows the EL spectra of devices 3 and 4 (Figure 2a, curve 3 and Figure 2b, curve 2, respectively). In both cases, the EL spectra contain no wide band around 560 nm and exhibit only one band in the blue region with the maximum at 471 nm (device 3) and 469 nm (device 4), which may be attributed mainly to the intrinsic emission of the Zn(PSA-BTZ)_2 _complex. Similar result on eliminating the exciplex EL by introducing the intermediate CBP layer is described below for Zn(POPS-BTZ)_2_-based OLEDs.

It should be noted that both NPD and PTA, as well as many other materials usually used to form the hole-transporting layer, are the derivatives of triarylamines. One may suppose that the interaction of the nitrogen atoms in the amino groups of the hole-transporting molecules and the amino groups of the zinc complexes (due to their spatial overlap) determines the exciplex formation in the studied systems. Evidence in favor of this supposition comes from our results on using other materials different from triarylamine derivatives for hole-transporting layers. Figure 2a, curve 4 shows the EL spectrum of device ITO/PEDOT:PSS/Zn(PSA-BTZ)_2_/Al:Ca (device 5) where the hole-transporting layer is presented by PEDOT:PSS, a hole injecting and transporting material which does not contain nitrogen atoms at all. This spectrum does not contain a wide band around 560 nm and exhibits only one band with a maximum at 466 nm, which is close to the Zn(PSA-BTZ)_2 _powder PL band (450 nm) and may be attributed mainly to the intrinsic emission of Zn(PSA-BTZ)_2 _complex. One may suppose that the formation of exciplex in this case is suppressed by the absence of nitrogen atoms in the hole-transporting layers.

Commonly, the reason for preventing the exciplex emission by changing the hole-transporting material is argued to be the relation between the energy levels of the donor and acceptor molecules. Materials like CBP with low highest occupied molecular orbital (HOMO) energy level are considered as appropriate ones [9,10,12,13]. Really, the HOMO level of CBP is 6.1 to 6.3 eV below vacuum level [23-25], which is appreciably lower than that of NPD (5.2 to 5.7 eV [24,26,27]). On the other hand, the highest occupied energy level of PEDOT:PSS is 5.2 eV below vacuum level [28], which does not differ from that of NPD. So, the fact that NPD produces exciplexes with the studied complexes and CBP and PEDOT:PSS do not may be explained not only by positions of energy levels but also by other reasons. Good spatial overlap of donor and acceptor molecular orbitals seems to be one of the most important factors promoting the formation of exciplexes.

From this point of view, molecules with amino groups are most appropriate for exciplex formation because of high electron density at nitrogen atoms. Zinc complexes studied in the present work contain amino groups bonded to metal atom and produce exciplexes in pair with triarylamine molecules NPD and PTA. Note that the analogs of our complexes containing oxygen atom bonded to metal such as Mq_3_, Znq_2_, Zn(BTZ)_2 _do not exhibit exciplexes in their EL spectra when triarylamine hole-transporting materials like NPD or TPD are used [1-6]. At the same time, the derivatives of Alq3 containing amino groups bonded to quinoline species exhibit EL exciplex bands for the devices with NPD [10].

### EL spectra of OLEDs based on Zn(TSA-BTZ)_2_

**Figure 3:**
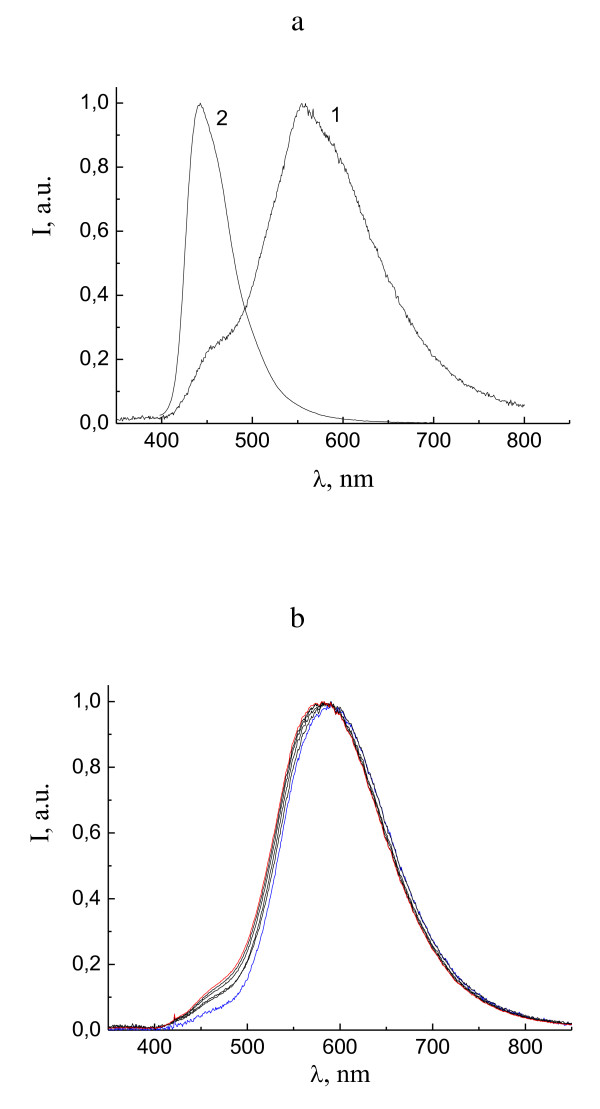
**Normalized EL spectrum of devices 6 and 7**. **(a) **Normalized EL spectrum of device 6, ITO/PTA/NPD/Zn(TSA-BTZ)_2_/Al:Ca, (1) and PL spectrum of Zn(TSA-BTZ)_2 _powder (2). **(b) **Normalized EL spectra of device 7, ITO/PTA/Zn(TSA-BTZ)_2_/Al:Ca, for bias voltages 3.5 (blue curve), 4.0, 4.5, 5.0, 5.5 (black curves), and 6.0 V (red curve).

Figure 3 shows the EL spectra of Zn(TSA-BTZ)_2 _in two electroluminescence devices: device 6, ITO/PTA/NPD/Zn(TSA-BTZ)_2_/Al:Ca, (Figure 3a) and device 7, ITO/PTA/Zn(TSA-BTZ)_2_/AlCa, (Figure 3b). The PL spectrum of Zn(TSA-BTZ)_2 _powder is shown for comparison (Figure 3b, curve 2). For both devices, intensive exciplex EL bands are observed in the yellow region with the maxima around 585 nm. Only a weak shoulder in the region of the intrinsic Zn(TSA-BTZ)_2 _emission at about 460 nm is observed. For device 7, the EL spectra are shown for different bias voltages from 3.5 to 6.0 V. The spectra are normalized to obtain equal intensities of exciplex bands for all voltages. A small continuous growth of intrinsic emission relative intensity is observed. A small blue shift of exciplex band maximum from 585 nm at 3.5 V to 575 nm at 6.0 V is also observed. This is in contrast with previously reported strong dependence of exciplex band positions on bias voltages [11].

### EL spectra of OLEDs based on Zn(POPS-BTZ)_2_

**Figure 4:**
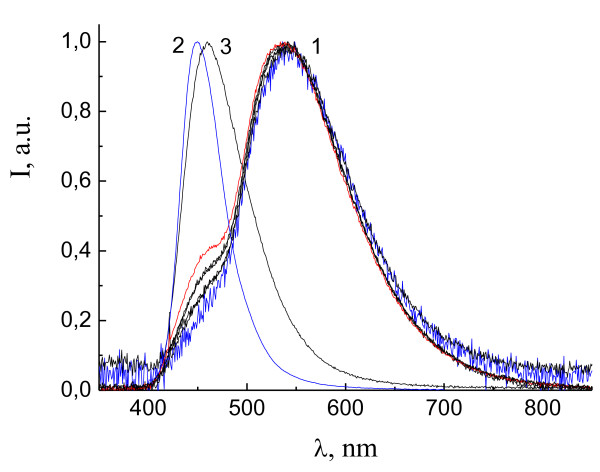
**Normalized EL spectra of device 8, ITO/PTA/NPD/Zn(POPS-BTZ)_2_/Al:Ca**. For bias voltages 4.0 (blue curve), 4.5, 5.0, 5.5 (black curves), and 6.0 V (red curve) (1). PL of Zn(POPS-BTZ)_2 _powder (2), and the EL spectrum of device 9, ITO/PTA/NPD/CBP/Zn(POPS-BTZ)_2_/Al:Ca, (3).

Figure 4 shows the EL spectra of Zn(POPS-BTZ)_2 _in ITO/PTA/NPD/Zn(POPS-BTZ)_2_/Al:Ca (device 8). The PL spectrum of Zn(POPS-BTZ)_2 _powder (curve 2) is shown for comparison. Strong exciplex band in the green region with the maximum at about 540 nm and shoulder at about 460 nm due to intrinsic emission of Zn(POPS-BTZ)_2 _is observed in the EL spectra. The normalized EL spectra are shown for different bias voltages from 4.0 to 6.0 V. A small continuous growth of intrinsic emission relative intensity and a small blue shift of exciplex band maximum from 545 nm at 4.0 V to 535 nm at 6.0 V are also observed.

Similar to the devices based on Zn(PSA-BTZ)_2_, the exciplex band can be eliminated by introducing the intermediate layer of CBP between NPD and Zn(POPS-BTZ)2. The EL spectrum of the device ITO/PTA/NPD/CBP/Zn(POPS-BTZ)_2_/Al:Ca (device 9) is shown in Figure 4 (curve 2). The exciplex band in the region of 540 nm is absent, and only the intrinsic emission of Zn(POPS-BTZ)_2 _at *λ*max = 460 nm is observed.

### EL spectra of OLEDs based on Zn(DFP-SAMQ)_2_

**Figure 5:**
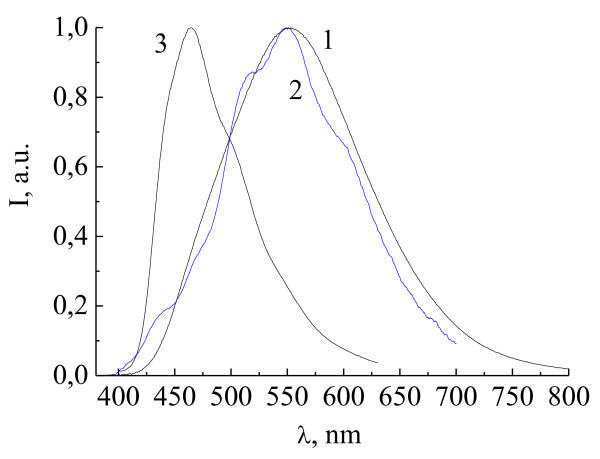
**EL spectra of devices 10, ITO/PTA/NPD/Zn(DFP-SAMQ)_2_/Al:Ca, (1) and 11, ITO/PTA/Zn(DFP-SAMQ)_2_/Al:Ca, (2)**. PL of Zn(DFP-SAMQ)_2 _powder (3).

Figure 5 shows the EL spectra of Zn(DFP-SAMQ)_2 _in the devices ITO/PTA/NPD/Zn(DFP-SAMQ)_2_/AlCa (device 10, curve 1) and ITO/PTA/Zn(DFP-SAMQ)_2_/AlCa (device 11, curve 2). For comparison, the PL spectrum of Zn(DFP-SAMQ)_2 _powder is shown (curve 3). Exciplex bands with maxima at about 560 nm are observed in the EL spectra; no intrinsic emission is observed.

### The PL spectra of the films containing blends of zinc complex and of hole-transporting material

One more evidence of the exciplex nature of long-wave bands in the EL spectra of zinc-chelate complexes with sulphanilamino-substituted ligands comes from their PL spectra in blends with hole-transporting materials. It was shown previously that the PL spectra taken from the layered structure exhibiting the exciplex EL OLEDs do not contain long-wave bands but only the intrinsic bands of components [21]. This is due to the extremely small thickness of the contacting interface of the two layers, which is responsible for EL. To observe the long-wave bands in PL, we prepared films containing blends of zinc complex and hole-transporting material. The films were prepared by casting from toluene solutions containing both components in appropriate concentrations. In such films, contacts between the two kinds of molecules take place in the whole volume of the film, unlike the bilayer OLED structure with very thin contact interface. The exciplex PL spectra of similar type mixed films were demonstrated previously [7].

**Figure 6:**
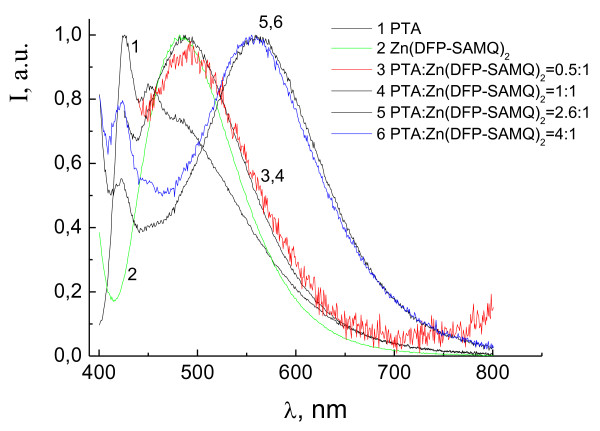
**PL spectra of the films containing PTA, Zn(DFP-SAMQ)_2 _and their blends**.

Figure 6 shows the PL spectra of the films containing PTA, Zn(DFP-SAMQ)_2 _and their blends. For the films with a relatively low fraction of PTA where PTA:Zn(DFP-SAMQ)_2 _= 0.5:1 and 1:1 (mass), the PL bands are close to that of Zn(DFP-SAMQ)_2 _with *λ*max = 490 nm (intrinsic emission). For the films with a higher PTA fraction where PTA:Zn(DFP-SAMQ)_2 _= 2.6:1 and 4:1 (mass), the exciplex PL band with *λ*max = 560 nm is observed. This result shows that the exciplex PL can be observed for donor-acceptor blends with proper relation between components, which guarantees large amount and good quality of intermolecular donor-acceptor contacts.

## Conclusions

The novel zinc-chelate complexes of sulphanilamino-substituted quinolines and benzothiazoles are proper materials for OLEDs, with efficient exciplex emission giving rise to white OLEDs and OLEDs of different colors including blue, green, and yellow. Exciplex emission can also be observed in the PL spectra of the films containing blends of zinc complex and hole-transporting material. Material of the hole-transporting layer is crucial for the efficiency of exciplex formation. Triarylamine derivatives seem to be the most proper materials for exciplex formation. This may be due not only to positions of energy levels but also to good spatial overlap and high electron density on amino groups of both zinc complex and triarylamine derivatives.

## Competing interests

The authors declare that they have no competing interests.

## Authors' contributions

MGK contributed to the redaction of the manuscript and in the design of the study. SSK and SLN carried out the preparation of the devices and measuring of their properties. NLS carried out the preparation of blended films and measuring of their spectral properties. IKY have synthesized the zinc complexes and PTA. All authors read and approved the final manuscript.

## References

[B1] Burrows PE Shen Z Bulovic V McCarty DM Forrest SR Cronin JA Thompson ME Relationship between electroluminescence and current transport in organic heterojunction light-emitting devices J Appl Phys 1996 79 7991 10.1063/1.362350

[B2] Burrows PE Sapochak LS McCatty DM Forrest SR Thompson ME Metal ion dependent luminescence effects in metal tris-quinolate organic heterojunction light emitting devices Appl Phys Lett 1994 64 2718 10.1063/1.111453

[B3] Tang CW Van Slyke SA Organic electroluminescent diodes Appl Phys Lett 1987 51 913 10.1063/1.98799

[B4] Hamada Y Sano T Fujita M Fujii T Nishio Y Shibata K Organic electroluminescent devices with 8-hydroxyquinoline derivative-metal complexes as an emitter Jpn J Appl Phys 1993 32 L514 10.1143/JJAP.32.L514

[B5] Hamada Y Sano T Fujii H Nishio Y Takahashi H Shihata K White light emitting material for organic electroluminescent devices Jpn J Appl Phys 1996 35 L1339 10.1143/JJAP.35.L1339

[B6] Tanaka H Tokito S Taga Y Okada A Novel metal-chelate emitting materials based on polycyclic aromatic ligands for electroluminescent devices J Mater Chem 1998 8 1999

[B7] Thompson J Blyt RIR Mazzeo M Anni M Gigli G Clinigolani R White light emission from blends of blue-emitting organic molecules: a general route to the white organic light-emitting diode? Appl Phys Lett 2001 79 560 10.1063/1.1388875

[B8] Cocchi M Virgili D Giro G Fattori V Marco PD Kalinowski J Shirtota Y Efficient exciplex emitting organic electroluminescent devices Appl Phys Lett 2002 80 2401 10.1063/1.1467614

[B9] Su WM Li WL Xin Q Su ZS Chu B Bi DF He H Niu JH Effect of acceptor on efficiencies of exciplex-type organic light emitting diodes Appl Phys Lett 2007 91 043508 10.1063/1.2762298

[B10] Liao S-H Shiu J-R Liu S-W Yeh S-J Chen Y-H Chen C-T Chow TJ Wu C-I Hydroxynaphthyridine-derived group III metal chelates: wide band gap and deep blue analogues of green Alq3 (Tris(8-hydroxyquinolate)aluminum) and their versatile applications for organic light-emitting diodes J Am Chem Soc 2009 131 763 10.1021/ja807284e 19093863

[B11] Itano K Ogawa H Shirota Y Exciplex formation at the organic solid-state interface: yellow emission in organic light-emitting diodes using green-fluorescent tris(8-quinolinolato)aluminum and hole-transporting molecular materials with low ionization potentials Appl Phys Lett 1998 72 636 10.1063/1.120826

[B12] Li G Kim CH Zhou Z Shinar J Okumoto K Shirota Y Combinatorial study of exciplex formation at the interface between two wide band gap organic semiconductors Appl Phys Lett 2006 88 253505 10.1063/1.2202391

[B13] Noda T Ogawa H Shirota Y A blue-emitting organic electroluminescent device using a novel emitting amorphous molecular material, 5,5'-bis(dimesitylboryl)-2,2'-bithiophene Adv Mater 1999 11 283 10.1002/(SICI)1521-4095(199903)11:4<283::AID-ADMA283>3.0.CO;2-V

[B14] Liu Y Guo J Zhang H Wang Y Highly efficient white organic electroluminescence from a double-lay device based on a boron hydroxyphenylpyridine complex Angew Chem Int Ed 2002 41 182 10.1002/1521-3773(20020104)41:1<182::AID-ANIE182>3.0.CO;2-B 12491480

[B15] Chao C-I Chen S-A White light emission from exciplex in a bilayer device with two blue light-emitting polymers Appl Phys Lett 1998 73 426

[B16] Tong QX Lai SL Chan MY Tang JX Kwong HL Lee CS Lee ST High-efficiency nondoped white organic light-emitting devices Appl Phys Lett 2007 91 023503 10.1063/1.2756137

[B17] Li M Li W Chen L Kong Z Chu B Li B Hu Z Zhang Z Tuning emission color of electroluminescence from two organic interfacial exciplexes by modulating the thickness of middle gadolinium complex layer Appl Phys Lett 2006 88 091108 10.1063/1.2181194

[B18] Yakushchenko IK Kaplunov MG Krasnikova SS Roshchupkina OS Pivovarov AP A New class of electroluminescent metal complexes based on quinoline ligands containing the sulfanylamino group Russ J Coord Chem 2009 35 312 10.1134/S1070328409040137

[B19] Kaplunov MG Yakushchenko IK Krasnikova SS Pivovarov AP Electroluminescent devices based on novel zinc complexes of sulphonylamino substituted heterocycles Mol Cryst Liq Cryst 2008 497 211

[B20] Kaplunov MG Yakushchenko IK Krasnikova SS Pivovarov AP Balashova IO Electroluminescent materials based on new metal complexes for organic light-emitting diodes High Energ Chem 2008 42 563 10.1134/S0018143908070199

[B21] Krasnikova SS Kaplunov MG Yakushchenko IK Exciplex electroluminescence spectra of organic light emitting diodes based on zinc complexes with sulfonylamino substituted ligands High Energ Chem 2009 43 536 10.1134/S0018143909070054

[B22] Yakushchenko IK Kaplunov MG Efimov ON Belov MY Shamaev SN Polytriphenylamine derivatives as materials for hole transporting layers in electroluminescent devices Phys Chem Chem Phys 1999 1 1783

[B23] Hill IG Rajagopai A Kahn A Energy-level alignment at interfaces between metals and the organic semiconductor 4,48-N, N8-dicarbazolyl-biphenyl J Appl Phys 1998 84 3236 10.1063/1.368477

[B24] Baldo MA Lamansky S Burrows PE Thompson ME Forrest SR Very high-efficiency green organic light-emitting devices based on electrophosphorescence Appl Phys Lett 1999 75 4 10.1063/1.124258

[B25] Adamovich V Brooks J Tamayo A Alexander AM Djurovich PI D'Andrade BW Adachi C Forrest SR Thompson ME High efficiency single dopant white electrophosphorescent light emitting diodes New J Chem 2002 26 1171 10.1039/b204301g

[B26] Brutting W Berleb S Muckl AG Device physics of organic light-emitting diodes based on molecular materials Org Electron 2001 2 1 36 10.1016/S1566-1199(01)00009-X

[B27] Lee M-T Yen C-K Yang W-P Chen H-H Liao C-H Tsai C-H Chen CH Efficient green coumarin dopants for organic light-emitting devices Org Lett 2004 6 1241 10.1021/ol049903d 15070307

[B28] Kim JY Kim M Kim HM Joo J Choi J-H Electrical and optical studies of organic light emitting devices using SWCNTs-polymer nanocomposites Opt Mater 2002 21 147

